# Allogeneic NKG2D CAR-T Cell Therapy: A Promising Approach for Treating Solid Tumors

**DOI:** 10.3390/biomedicines13092314

**Published:** 2025-09-22

**Authors:** Sabir A. Mukhametshin, Elvina M. Gilyazova, Damir R. Davletshin, Irina A. Ganeeva, Ekaterina A. Zmievskaya, Vitaly V. Chasov, Alexsei V. Petukhov, Aigul Kh. Valiullina, Sheila Spada, Emil R. Bulatov

**Affiliations:** 1 Institute of Fundamental Medicine and Biology, Kazan Federal University, 420008 Kazan, Russia; 2 Laboratory of Molecular Oncology, National Laboratory Astana, Astana 010000, Kazakhstan; 3 Department of Biomedical Sciences, School of Medicine, Nazarbayev University, Astana 010000, Kazakhstan; 4 Tumor Immunology and Immunotherapy Unit, IRCCS-Regina Elena National Cancer Institute, 00144 Rome, Italy; 5 Shemyakin-Ovchinnikov Institute of Bioorganic Chemistry, Russian Academy of Sciences, 117997 Moscow, Russia

**Keywords:** allogeneic CAR-T therapy, CRISPR/Cas9, cancer, adoptive cell therapy, NKG2D receptor

## Abstract

Chimeric Antigen Receptor (CAR)-T cell therapy has transformed the treatment landscape of cancer, yet major challenges remain in enhancing efficacy, reducing adverse effects, and expanding accessibility. Autologous CAR-T cells, derived from individual patients, have achieved remarkable clinical success in hematologic malignancies; however, their highly personalized nature limits scalability, increases costs, and delays timely treatment. Allogeneic CAR-T cells generated from healthy donors provide an “off-the-shelf” alternative but face two critical immune barriers: graft-versus-host disease (GvHD), caused by donor T-cell receptor (TCR) recognition of host tissues, and host-versus-graft rejection, mediated by recipient immune responses against donor HLA molecules. Recent advances in genome engineering, particularly Clustered Regularly Interspaced Short Palindromic Repeats (CRISPR)/Cas9, allow precise modification of donor T cells to overcome these limitations. For example, TRAC gene knockout eliminates TCR expression, preventing GvHD, while disruption of HLA molecules reduces immunogenicity without impairing cytotoxicity. Beyond hematologic cancers, CRISPR-edited allogeneic CAR-T cells targeting the NKG2D receptor have shown promise in preclinical studies and early-phase trials. NKG2D CAR-T cells recognize stress ligands (MICA/B, ULBP1–6) expressed on over 80% of diverse solid tumors, including pancreatic and ovarian cancers, thereby broadening therapeutic applicability. Nevertheless, the genomic editing process carries risks of off-target effects, including potential disruption of tumor suppressor genes and oncogenes, underscoring the need for stringent safety and quality control. This review examines the distinguishing features of allogeneic versus autologous CAR-T therapy, with a particular focus on NKG2D-based allogeneic CAR-T approaches for solid tumors. We summarize current strategies to mitigate immune barriers, discuss practical manufacturing challenges, and analyze available clinical data on NKG2D CAR-T trials. Collectively, these insights underscore both the promise and the hurdles of developing safe, universal, and scalable allogeneic CAR-T therapies for solid malignancies.

## 1. Introduction

Chimeric antigen receptor (CAR)-T cell therapy represents a major breakthrough in modern oncology, demonstrating unprecedented efficacy in the treatment of relapsed and refractory hematologic malignancies. By redirecting patient-derived T lymphocytes against specific tumor antigens, CAR-T therapy has achieved durable remissions in diseases such as B-cell acute lymphoblastic leukemia and large B-cell lymphoma. Despite these successes, the application of CAR-T therapy to solid tumors remains limited due to barriers such as tumor heterogeneity, an immunosuppressive tumor microenvironment, and physical obstacles to T-cell trafficking and infiltration. To address these limitations, allogeneic CAR-T cell therapy has emerged as a promising alternative to conventional autologous approaches. Unlike autologous CAR-T cells, which rely on patient-derived T lymphocytes, allogeneic CAR-T cells are generated from healthy donors. This strategy provides several key advantages, including standardized large-scale manufacturing, immediate availability of therapeutic products, and the ability to select high-quality donor T cells with superior proliferative and cytotoxic potential. These attributes make allogeneic CAR-T cells an attractive “off-the-shelf” option that could improve accessibility and reduce treatment delays. However, translation of allogeneic CAR-T therapy into the clinic is complicated by major immune compatibility barriers. Donor T cells can elicit graft-versus-host disease (GvHD) through recognition of host antigens, while the host immune system can mount host-versus-graft (HvG) rejection against donor cells, thereby limiting persistence and efficacy [1]. To overcome these risks, targeted genome-editing strategies have been developed. Disruption of the T-cell receptor alpha constant (TRAC) gene eliminates endogenous TCR expression and prevents GvHD, while knockout of β2-microglobulin (B2M) reduces expression of major histocompatibility complex (MHC) class I molecules, thereby mitigating HvG-mediated rejection [2]. Together, these genetic interventions form the foundation for the development of safe, universal, and scalable allogeneic CAR-T platforms.

## 2. CAR-T Therapy as an Example of Adoptive Cell Therapy for Cancer Diseases

Cancer is a complex disease driven by accumulation of critical mutations in genes, culminating in oncogenic transformation and uncontrollable proliferation of malignant cells. Over the past centuries, cancer therapies such as surgery, chemotherapy, and radiation therapy have been developed. Despite the success achieved, the treatment of patients suffering from cancer remains difficult. Tumor heterogeneity, metastasis, and the development of resistance to antitumor therapeutics reduce the effectiveness of standard treatments such as chemotherapy and radiotherapy. Conventional therapy in a number of patients causes alternating periods of remission and relapse, which can lead to the development of signs of chronic cancer [3]. Due to the high toxicity of chemotherapy and radiotherapy, as well as the limited positive response to conventional treatments, the search for new approaches to cancer therapy is urgent.

In recent years, immunotherapy has become one of the main types of therapy for cancer patients. The result of immunotherapy is the activation of mechanisms that destroy tumor cells using chemokines, cytokines, and cell-mediated cytotoxicity [4,5]. Existing varieties of cancer immunotherapy include monoclonal antibodies, immune checkpoint inhibitors, cytokines, vaccines, and adoptive cell transfer [6].

The main levers of the immune system against pathogens and tumor cells are innate and adaptive immunity. The immune system has three main functions in cancer prevention. First, it protects the body from viral oncogenesis. Second, through timely elimination of pathogens, the immune system eliminates the inflammatory microenvironment that promotes tumorigenesis. Third, the immune system is able to identify and eliminate tumor cells based on the expression of tumor-specific antigens. However, tumor cells are able to evade the immune response and proliferate, particularly when immune defenses are suppressed [7].

T lymphocytes are a key component of the adaptive immune response. Recently, strategies of genetic modification of T cells by changing the specificity of the T-cell receptor (TCR) or introducing antibody-like recognition domains in the form of chimeric antigen receptors have made significant progress [8].

In the case of adoptive T-cell therapy, the patient’s T lymphocytes are isolated and modified, then infused back to the patient. The three main types of adoptive T-cell therapy include tumor-infiltrating lymphocytes (TILs), modified CAR-T cells and TCR-T cells [9].

TIL therapy involves isolation of tumor-infiltrating lymphocytes from solid tumors and subsequent expansion for several weeks ex vivo under sterile conditions using a cytokine cocktail to obtain a finished cell preparation. Expansion of TILs requires a large amount of time, which worsens the availability of the drug to the patient [10].

T-cell receptor (TCR)-engineered T-cells (TCR-T) utilize TCRs that are found on native T-cells to confer specificity instead of antibody-based CARs. TCRs can be isolated from tumor-reactive T cells and further modified to improve expression and effector functions. TCRs can recognize both surface and intracellular antigens including neoantigens (neoAgs) that arise from mutations and are specific to tumor cells. A disadvantage of using TCR-T is that TCRs are restricted to the patient’s MHC molecules, so any TCR can only be used to treat patients with the appropriate MHC haplotype [11].

CAR-T therapy (chimeric antigen receptor T-cells) is a method of cellular immunotherapy in which T-lymphocytes are modified with a chimeric antigen receptor to specifically recognize and eliminate tumor cells. The chimeric antigen receptor consists of four major components: an extracellular antigen-binding domain, a hinge region, a transmembrane domain, and an intracellular region consisting of several domains depending on the generation of the CAR. The extracellular domain of the chimeric antigen receptor is a complex of heavy and light chains of an antibody specific to the tumor antigen (single chain variable fragment). The intracellular domains (CD3ζ and CD28) of the CAR provide activation and costimulatory signals upon tumor antigen recognition, and another domains trigger additional signaling cascades depending on the CAR generation: 4-1BB (3rd generation) improves persistence and cytotoxicity of CAR-T cells, NFAT (4th generation) provides IL-12 synthesis for activation of neighboring T-lymphocytes, IL-2β (5th generation) triggers JAK/STAT3 signaling cascade to improve proliferation and persistence of CAR-T cells. A feature of the chimeric antigen receptor is the ability to recognize tumor antigens in an MHC-independent manner, which enables T-lymphocyte activation and subsequent tumor lysis by lytic granules (perforin and granzyme) [12,13].

Recent significant breakthroughs in CAR-T therapy have improved the process of generating and utilizing CAR-T lymphocytes. For example, the use of mRNA molecules encoding the sequence of a chimeric antigenic receptor ensures temporary expression of CAR, unlike lentiviral transduction, and also reduces the risk of mutations caused by the integration of the CAR cassette into the genome. Lipid nanoparticles conjugated with T-lymphocyte-specific antibodies are a promising carrier of mRNA molecules for targeted delivery. This approach also allows the delivery of CAR mRNA to T-lymphocytes directly in the patient’s body (in vivo CAR-T), which is designed to simplify the process of CAR-T lymphocyte production and distribution to the patient (no need for T-lymphocyte apheresis, transduction, and cell mass production in vitro) [14,15].

The combination of oncolytic viruses and CAR-T lymphocytes is a promising approach to the treatment of solid tumors. Oncolytic viruses cause lysis of tumor cells, resulting in the release of tumor antigens that activate CAR-T cells. Additionally, oncolytic viruses are able to alter the cellular composition of the tumor microenvironment, which leads to a decrease in local immunosuppression and a more active cytotoxic response of CAR-T lymphocytes [16].

The loss of tumor antigen and subsequent recurrence of the disease after CAR-T therapy is a significant problem. To overcome this, it is proposed to use bispecific CAR-T lymphocytes capable of recognizing several antigenic domains or individual target molecules on the surface of tumor cells. The following approaches are used to ensure dual recognition of tumor antigens: T-cell transduction with two separate CAR constructs, T-cell transduction with a single construct with two CAR matrices, the introduction of two populations of CAR-T cells with different specificity, as well as the use of modified CARs with multiple antigen-binding domains (tandem CAR). The technology of bispecific CAR-T lymphocytes made it possible to increase the specificity of tumor cell damage and ensure controlled activation of CAR-T lymphocytes, which reduces the severity of cytokine release syndrome and slows down the depletion of CAR-T cells, respectively. CAR-T lymphocytes with double and triple specificity to tumor antigens have shown promising results in clinical trials against B-cell tumor diseases, including multiple myeloma [17,18]. Nevertheless, the design of CAR cassettes with limited capacity of delivery systems is the main difficulty of this technology and requires the search for compromise solutions, for example, the removal of the costimulating domain CAR 4-1BB [19].

Adoptive immunotherapy using CAR-T cells has emerged as a promising therapeutic option for several types of malignancies. Impressive results with CAR-T cells targeting CD19 and BCMA have led to FDA and EMA approval of several CAR-T cell products for the treatment of refractory acute lymphoblastic leukemia from refractory progenitor cells and large B-cell lymphoma: Kymriah, Yescarta, Tecartus, Breyanzi, Abecma, Carvykti and Carteyva [20,21].

## 3. CAR-T Cell Therapy: Autologous and Allogeneic Approaches

There are two ways of making CAR-T cells, depending on the source of the T lymphocytes. Most CAR-T lymphocytes are taken from the patient’s own immune cells and injected back into the patient—these CAR-T cells are called autologous. They are widely used in the treatment of hematological tumors. A more advanced technology of CAR-T cell production is based on donor-derived T lymphocytes—allogeneic CAR-T therapy. Allogeneic CAR-T cells have many advantages over autologous CAR-T therapy [22,23].

The efficacy of autologous CAR-T cell therapy is undeniable, but the patient’s own T cells are not an ideal material for cell therapy. A patient’s clinical response is related to the expansion of CAR-T cells in the body, which in turn is related to the in vitro expansion rate of CAR-T cells [24]. Previous studies have shown that autologous CAR-T cells have a lower expansion rate compared to cell material from healthy donors [25]. Another limitation of autologous CAR-T cell therapy is the negative relationship between the amount of prior therapy and CAR-T cell expansion [26]. Preclinical studies have shown that autologous CAR-T cells have lower antitumor activity than a CAR-T drug made from donor-derived T lymphocytes [27]. In this type of therapy, each patient requires a personalized production cycle, whereas in the production of an allogeneic CAR-T cell product, cells can be harvested in large batches that can potentially be used to treat 100 or more patients. Autologous CAR-T cell therapy can also be hampered by supply failures, as some patient cells are not capable of expansion and may have characteristics that exceed quality control targets. Disease progression while waiting for CAR-T cells to be manufactured is a serious problem, resulting in approximately 10–23% of patients not receiving treatment [28]. Quality control of the product itself can also be challenging, as each sample requires multiple tests, the number of which increases as the product design becomes more complex [29].

Given the significant problems with expansion, high cost, difficulty in obtaining and the possibility of producing ready-to-use products from autologous CAR-T cells, allogeneic CAR-T cell drugs represent a more promising method of immunotherapy for tumor diseases (Table 1).

Allogeneic CAR-T lymphocyte technology allows manufacturers to control the age of the donor for cellular drug production. T-lymphocytes from young donors show lower expression of exhaustion markers and longer-term cytotoxicity against tumor cells [20,30]. An important advantage of allogeneic CAR-T therapy is the scalability of the manufacturing process, which allows multiple patients to be treated from a single production batch. Scaling up production reduces the waiting time to start treatment and eliminates the need for interim therapy. Allogeneic CAR-T cell therapy enables the creation of ready-to-use drug banks, which simplifies logistics and increases the speed of therapeutic delivery to the patient [31,32]

Despite the advantages of allogeneic CAR-T cell therapies, additional modifications are required to prevent the side effects of allogeneic CAR-T therapy: graft-versus-host disease and graft rejection syndrome, potentially life-threatening conditions for the patient [33].

**Table 1:** Comparison of characteristics of allogeneic and autologous CAR-T cells [34,35].

Characteristics	Autologous CAR-T Cells	Allogeneic CAR-T Cells
Source of T cells	Patient	Healthy donor
Manufacturing process	Characteristics of CAR-T cells vary depending on the patient’s condition and previous therapy; long interval between leukapheresis and infusion of CAR-T cells; logistical difficulties	Possibility of standardizing the characteristics of CAR-T lymphocytes; large-scale industrial process for obtaining large numbers of CAR-T lymphocytes from a single donor; immediate availability of the drug for patient treatment.
Persistence in the patient’s body	From a few months to several years	From a few weeks to several months
Main disadvantages and side effects	Cytokine release syndrome; neurotoxicity; immune effector cell-associated neurotoxicity syndrome; long-term side effects (B-cell aplasia for anti-CD19 CAR-T cells)	Cytokine release syndrome; “off-target” mutations; graft-versus-host disease; rejection of modified CAR-T cells
Main disadvantages and side effects	Cytokine release syndrome; neurotoxicity; immune effector cell-associated neurotoxicity syndrome; long-term side effects (B-cell aplasia for anti-CD19 CAR-T cells)	Cytokine release syndrome; “off-target” mutations; graft-versus-host disease; rejection of modified CAR-T cells
Targeted tumor type	Hematological malignancies; solid malignancies	Hematological malignancies; solid malignancies
Reinfusion of CAR-T cells	Limited by the number of CAR-T cells	Not limited by number of CAR-T cells; increased risk of alloimmunization with each reinfusion
Cost	High cost ($300,000–475,000 per dose)	The ability to manage prices while developing the manufacturing process ($4000–10,000 per dose)

Allogeneic anti-CD19 CAR-T lymphocytes have been shown to be a rapidly accessible therapy, with a single dose sufficient to achieve a therapeutically significant effect. Data from phase I clinical trials of anti-CD19 AlloCAR T cells (ALLO-501—NCT03939026) and its successor ALLO-501A (NCT04416984) demonstrated a manageable safety profile with no dose-limiting toxicity and tumor cell efficacy [36].

Allogeneic donor anti-CD19 CAR-T lymphocytes are an effective and safe therapy for patients with relapsed/refractory B-cell acute lymphoblastic leukemia after allogeneic hematopoietic stem cell transplantation. Eight patients (72.7%) achieved a morphological remission. Seven patients (63.6%) had minimal residual disease—negative remission. The duration of ongoing complete remission (CR) reached 22 months in 2 patients. Median overall survival was 9 months (range 2–22 months). Only one patient experienced grade 1 acute graft-versus-host disease. Two patients (18.2%) developed grade 3/4 cytokine release syndrome [37].

## 4. Problems with the Interaction of Allogeneic CAR-T Cells with the Host Immune System

Autologous CAR-T lymphocytes are made from the patient’s own T lymphocytes. They express antigens specific to the patient’s body and are recognized by the immune system as “their own”. Allogeneic CAR-T cells are based on donor T-lymphocytes, which have different surface antigens, including different HLA complexes. Therefore, when allogeneic CAR-T lymphocytes are injected into the patient’s body, side effects specific to allogeneic CAR-T therapy may occur: graft-versus-host disease and graft rejection syndrome [2,34].

**Figure 1:**
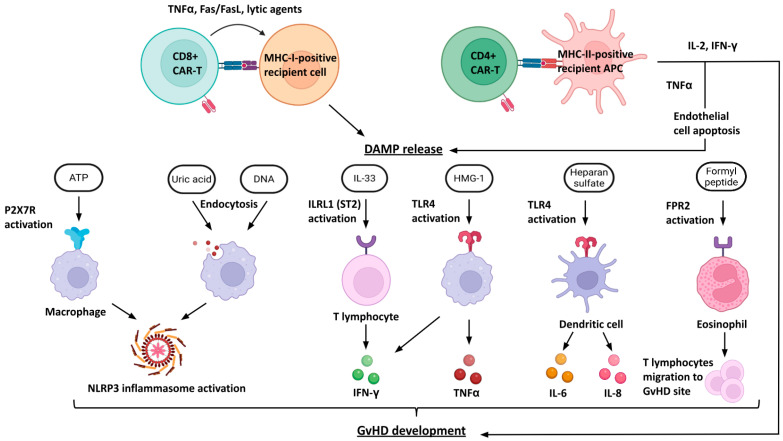
Mechanisms underlying graft-versus-host disease (GvHD) induced by allogeneic CAR-T cell infusion. Allogeneic CAR-T cells interact with host antigen-presenting cells (APCs) expressing MHC class I or II molecules, leading to activation of donor-derived CD8+ and CD4+ CAR-T lymphocytes through their residual T-cell receptor (TCR) complexes. Activated CAR-T cells release pro-inflammatory cytokines (e.g., TNFα, IFNγ, IL-2) and cytotoxic mediators (Fas/FasL, perforin, granzymes), resulting in direct host cell damage and endothelial apoptosis. Cellular injury promotes the release of damage-associated molecular patterns (DAMPs), including ATP, uric acid, DNA, IL-33, HMGB1, heparan sulfate, and formyl peptides. These DAMPs activate innate and adaptive immune pathways via specific receptors: P2X7R on macrophages (triggering NLRP3 inflammasome activation), ILRL1 (ST2) on T lymphocytes (inducing IFNγ release), and TLR4 on macrophages and dendritic cells (driving TNFα, IL-6, and IL-8 production). Additionally, formyl peptide receptor 2 (FPR2) activation on eosinophils promotes T-cell recruitment to inflammatory sites. Collectively, these cascades amplify inflammation, recruit effector immune cells, and drive the clinical manifestation of GvHD. Abbreviations: CD, cluster of differentiation; IL, interleukin; IFN, interferon; TNF, tumor necrosis factor; ATP, adenosine triphosphate; P2X7R, purinergic receptor P2X7; NLRP3, nucleotide-binding oligomerization domain-like receptor family pyrin domain containing 3; ILRL1, interleukin-1 receptor-like 1; HMGB1, high mobility group box 1; FPR2, formyl peptide receptor 2.

Graft-versus-host disease is mediated by the T cell receptor complex of infused cells, which recognizes the major histocompatibility complex class I (MHC-I) peptide repertoire of the host as foreign (Figure 1). During maturation in the thymus, CD8+ T cells become tolerized to their own MHC-I presented proteins. Donor CD8+ CAR-T cells are able to recognize multiple peptide-HLA-I complexes in the recipient, leading to the activation of CAR-T lymphocytes. This results in a cytotoxic response mediated by TNFα expression, perforin and granzyme release [38]. Donor CD4+ CAR-T lymphocytes are able to interact with MHC class II APCs of the recipient, leading to the synthesis of pro-inflammatory cytokines IL-2 and IFN-γ, further exacerbating GvHD. In addition, TNFα is synthesized, mediating endothelial cell destruction [39]. Thus, CD8+ and CD4+ CAR-T lymphocytes mediate the release of DAMPs into the extracellular space. ATP, uric acid and DNA molecules mediate NLRP3 inflammasome activation in macrophages. IL-33 interacts with the ST2 receptor (ILRL1) on the surface of recipient T lymphocytes, leading to the production of the pro-inflammatory IFN-γ [40]. High-mobility group protein B1 (HMGB-1) interacts with TLR4 on the surface of macrophages, resulting in the synthesis of IFN-γ and TNFα. Heparan sulfate, activating TLR4 of dendritic cells, induces the synthesis of IL-6 and IL-8. Finally, formyl peptide through activation of the corresponding receptor of eosinophils leads to migration of recipient T-lymphocytes to the focus of GvHD, intensifying inflammation [41].

Thus, DAMPs released as a result of activation of allogeneic CAR-T lymphocytes lead to the development of inflammation via recipient immunity mechanisms [42,43].

Several approaches have been developed to avoid GvHD caused by allogeneic CAR-T cells. The most obvious is to use a donor with a fully matched human leukocyte antigen (HLA), but given the low probability of finding a match by chance (e.g., 1 in 10,000), this would require a large bank of cell products, eliminating many of the benefits of “off-the-shelf” therapy [44]. Other routes include genetic knockout or knockdown of TCR and MHC-I genes, use of cells with known TCR specificity, or use of cell types that do not express the TCR alpha/beta complex capable of mediating GvHD [45].

The consequence of GvHD is the development of a shock state associated with the activation of allogeneic CAR-T cells upon recognition of recipient cell antigens and the release of large amounts of pro-inflammatory molecules (IL-1, IL-17, IL-21 and IL-22, TNF-α). Donor T cells recognize host alloantigens and become activated. Th1 cells are considered to be the main initiators of this process [46]. Donor T cells, which preferentially differentiate into Th1 cells and produce pro-inflammatory cytokines mediate GvHD [47]. As a result of the immune response of CAR-T lymphocytes to the recipient cells and subsequent immunosuppressive therapy, cytopenias develop, leading to a deficiency of immunocompetent cells in the patient’s body. Weakened immunity increases the risk of cancer recurrence and the likelihood of infection. Migration of allogeneic CAR-T lymphocytes to organs can lead to multiple organ failure and patient death [48].

**Figure 2:**
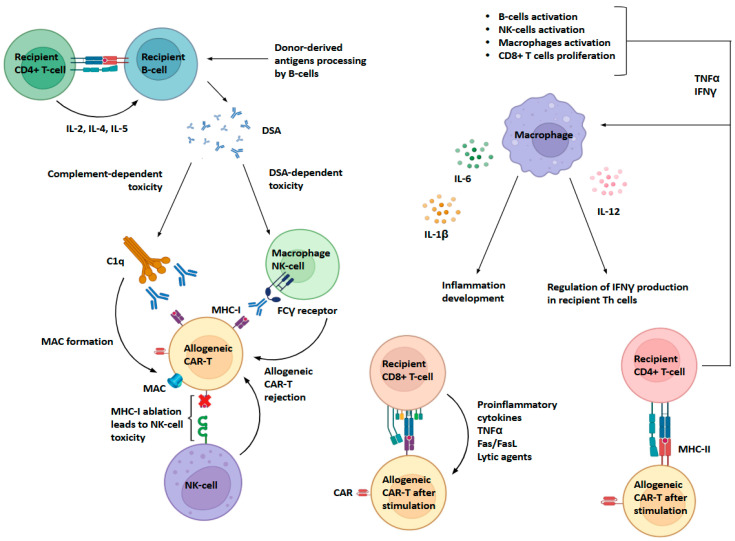
Mechanisms of allogeneic CAR-T cell rejection by the host immune system. Rejection of allogeneic CAR-T lymphocytes occurs through both antibody-dependent and cell-mediated mechanisms. Donor-derived antigens are recognized and processed by recipient B cells, leading to the generation of donor-specific antibodies (DSA). These antibodies contribute to CAR-T elimination through complement-dependent cytotoxicity, initiated by C1q binding and subsequent membrane attack complex (MAC) formation, as well as through Fcγ receptor-mediated phagocytosis and cytotoxicity by macrophages and NK cells. In parallel, direct allorecognition by recipient CD8+ and CD4+ T cells against MHC molecules on donor CAR-T cells induces secretion of TNFα, IFNγ, and other pro-inflammatory cytokines, along with Fas/FasL- and granzyme-mediated killing. Furthermore, NK cells can mediate cytotoxicity when donor CAR-T cells lack MHC-I expression (“missing-self” recognition). Activated macrophages amplify rejection by secreting IL-1β, IL-6, and IL-12, which promote inflammatory cascades, enhance Th cell IFNγ production, and support expansion of recipient immune subsets. Collectively, these mechanisms eliminate allogeneic CAR-T cells, suppress antitumor efficacy, and drive systemic inflammatory responses, including cytokine release syndrome (CRS), overlapping mechanistically with graft-versus-host disease (GvHD). Abbreviations: CD, cluster of differentiation; IL, interleukin; DSA, donor-specific antigen; C1q, complement component 1q; MAC, membrane attack complex; CAR, chimeric antigen receptor; NK, natural killer cell; TNF, tumor necrosis factor; IFN, interferon; Th, T helper.

Rejection syndrome in the case of infusion of allogeneic CAR-T lymphocytes develops by mechanisms similar to tissue graft rejection syndrome, but with limitations in the form of transfer of exclusively T-lymphocytes (Figure 2). Thus, direct cytotoxicity is provided when CD8+ T lymphocytes of the recipient recognize donor antigens as part of MHC class I on the surface of allogeneic CAR-T lymphocytes. This leads to the synthesis of pro-inflammatory cytokines by the recipient’s killer T cells, as well as to the expression of lytic substances (for example, perforin and granzyme). In addition, rejection of allogeneic CAR-T lymphocytes by CD8+ recipient T cells is ensured by the interaction of the Fas receptor and its ligand [49].

Simultaneously, CD4+ T lymphocytes of recipient are able to recognize antigens of allogeneic CAR-T lymphocytes in the composition of class II MHC, which is expressed on CAR-T lymphocytes after activation for lentiviral transduction. The result of this interaction is the synthesis of TNFα and IFNγ by activated CD4+ T-lymphocytes of the recipient, which leads to the complex activation of immune cells of the recipient: B lymphocytes, NK lymphocytes, CD8+ T lymphocytes and macrophages. The macrophage response to stimulation leads to the synthesis of cytokines IL-6 and IL-1β, which increases general inflammation. The synthesis of IL-12 by macrophages, in turn, regulates the expression of IFNγ by activated CD4+ recipient T cells. At the same time, IFNγ triggers a macrophage-mediated delayed-type hypersensitivity reaction that increases rejection [50,51].

In the process of direct allorecognition, allogeneic CAR-T lymphocytes are destroyed, which is accompanied by the release of donor antigens into the extracellular space. Recipient B-lymphocytes interact with donor antigens using the B-cell receptor and internalize them via endocytosis. As a result, donor antigens are delivered to intracellular compartments, where they bind to MHC class II molecules. Thus, B-lymphocytes express donor antigens, activating CD4+ T-cells of the recipient. The result of this interaction is the synthesis of donor-specific antibodies, which are involved in further rejection mechanisms [52].

One of the rejection mechanisms modulated by donor-specific antibodies involves activation of the complement system via the antibody-mediated pathway via factor C1q and subsequent formation of the membrane attack complex (MAC), disrupting the normal process of ion diffusion across the membrane, which leads to cell death [53]. Another mechanism involves targeting macrophages and NK cells through antibody-mediated interaction with allogeneic CAR-T cells using the FCγ receptor. In the case of natural killers, lytic granules (perforin and granzyme) are synthesized, which leads to rejection of allogeneic CAR-T lymphocytes. Activation of macrophages leads to the synthesis of pro-inflammatory cytokines (including IL-6, TNF-α, etc.) and rejection of allogeneic CAR-T lymphocytes, including the synthesis of reactive oxygen species and phagocytosis [54]. In the process of producing allogeneic CAR-T lymphocytes, it is necessary to eliminate MHC class I from the cell surface. The complete absence of MHC class I molecules leads to the development of NK cell cytotoxicity, since the major histocompatibility complex class I acts as an inhibitor of NK cell activation. Thus, when obtaining allogeneic CAR-T lymphocytes, it is necessary to take this aspect into account and carry out additional (overexpression of HLA-E) or alternative cell modifications (knockout or knockdown of HLA-A and HLA-B) [55].

The consequences of graft rejection syndrome are similar to those of GvHD. Activation of immunocompetent cells of the recipient leads to the release of cytokines and pro-inflammatory molecules to eliminate allogeneic CAR-T lymphocytes from the body—the patient develops shock, intoxication with cell apoptosis products and a number of pro-inflammatory molecules [56]. It is worth mentioning the clinical cases in which a patient undergoes hematopoietic stem cell transplantation from the same HLA-mismatched donor before infusion of allogeneic CAR-T cells [57]. In addition, it is proposed to use pluripotent stem cells from healthy donors with common HLA types as a source of T-lymphocytes to eliminate the need for knockout of HLA complexes in the manufacture of CAR-T cells [58].

To reduce the risk of rejection, it has been proposed to make CAR-T preparations based on cells from donors with rare HLA alleles. However, methods based on genome editing of CAR-T cells seem to be the most promising [59].

## 5. Genome Editing Techniques to Generate Universal and Safe CAR-T Cells

Modification of CAR-T cell surface molecules and therapeutic modalities may help to control the severity of GvHD and graft rejection syndrome.

In the clinic, immunosuppressive chemotherapy is used to reduce the risk of developing GvHD and graft rejection syndrome prior to infusion of allogeneic CAR-T lymphocytes. Chemotherapeutic and targeted drugs reduce the efficacy of the immune response and the proliferative capacity of both the patient’s own T lymphocytes and the injected CAR-T cells. Therefore, the use of modified allogeneic CAR-T cells, which can reduce the effects of graft rejection and alloreactivity, seems more appropriate [22].

Genome editing techniques are being used to improve the safety and manage the side effects of CAR-T therapy: ZFN (zinc finger nuclease), TALEN (transcription activator-like effector nuclease) and CRISPR/Cas9 (clustered regularly interspaced short palindromic repeats). ZFN is a dimer of 3–6 individual zinc fingers and the nuclease domain of the FokI restriction enzyme. The same nuclease domain is used by the TALEN system in complex with the DNA binding domain TAL of the TALE protein found in plants [60]. CRISPR/Cas9 is a genome editing system whose main structural elements are the guide RNA for target recognition and the Cas9 nuclease [61]. The aforementioned genome editing systems (Table 2) share a common mechanism of action: recognition of the target sequence in the DNA strand by the complex leads to the formation of a double-strand break. Such disruption of the double-stranded DNA structure leads to the mobilization of repair systems. Double-strand breaks can be repaired by two mechanisms: homologous end joining, based on the template of DNA introduced into the cell, and non-homologous end joining. Non-homologous end joining ensures the formation of deletions or insertions at the knockout site, leading to disruption of the gene structure and prevention of its expression. Thus, knocking out the genes responsible for the development of adverse effects of allogeneic CAR-T therapy can increase the safety of the cellular product and reduce the use of a large number of drugs to manage side effects. As a result, the patient’s exposure to drug metabolites is reduced [62].

To prevent graft rejection syndrome, allogeneic CAR-T lymphocytes should be depleted of HLA-I, which presents intracellular antigens of donor cells. The main target of editing in this case is the HLA class I beta-2-microglobulin (*B2M*) gene. Aberrations in the expression of this gene lead to disruption of the major histocompatibility complex class I (HLA-A, -B, -C, -E, -F and -G) and termination of the presentation of donor antigens on the surface of the allogeneic CAR-T lymphocyte. This approach prevents the allogeneic CAR-T cells from being recognized by the patient’s T lymphocytes as HLA-matched foreign cells. On the other hand, the absence of HLA-I increases the risk of rejection by natural killer cells. On the surface of NK cells are killer-cell immunoglobulin-like receptors (KIRs), whose ligands are molecules of the major histocompatibility complex class I. The lack of interaction between KIR and ligand triggers the process of NK cell activation. Allogeneic CAR-T lymphocytes lacking HLA-I require additional genomic modifications, for example, overexpression of the E and G subunits of HLA-I to prevent NK cell activation HLA-C and HLA-E subunits are also involved in providing T-cell resistance to NK cells [64,65].

In addition, knocking out the *HLA-A* and *HLA-B* genes in donor T cells while retaining HLA-C allows partial editing and reduction in HLA expression. This approach not only reduces the recognition of donor cells by host T cells but also attenuated NK cell-mediated rejection. The knockout of *B2M*/*HLA-A* genes in universal CAR-T cells with overexpression of HLA-E and HLA-G shows significantly increased persistence and efficacy in preclinical and clinical studies [66,67].

The main alteration that limits the development of GvHD by allogeneic CAR-T lymphocytes is the impaired expression of the T cell receptor (TCR). The TCR is a heterodimer consisting of two parts, the alpha (TCRα) and beta (TCRβ) subunits. Both subunits are required for T cell receptor expression. The TCR alpha subunit is encoded by a single gene (*TRAC*), whereas the beta subunit is encoded by two genes. Therefore, TRAC is the most promising gene for knockout to inhibit TCR expression [68].

One of the first technologies to inhibit TCR expression in allogeneic CAR-T cells is based on the interaction of TALEN restriction enzymes with the gene encoding the alpha subunit of the T-cell receptor TRAC. The efficiency of TCR inhibition by this method was 78% [55]. Subsequent attempts were made to knock out the *TRAC* gene using CRISPR/Cas9 and ZFN—the knockout efficiency was 70 and 60%, respectively [69]. *Gilham* and colleagues developed an approach to disrupt TCR function based on competitive inhibition of the TCR-CD3ζ component using a truncated dominant-negative CD3ζ protein (T-cell receptor inhibitor molecule—TIM). TIM interferes with TCR signaling and thereby reduces the risk of developing GvHD [70]. *Razeghian* and colleagues knocked out the *TRAC*, *B2M*, *PD-1* (programmed death receptor 1) and *CTLA-4* (cytotoxic T-lymphocyte associated protein 4) genes of allogeneic CAR-T lymphocytes. Knocked out cells exhibited cytotoxicity against tumor cells during in vivo and in vitro experiments without GvHD manifestation. Knockout of *PD-1*, *TRAC*, and *B2M* genes improves the cytotoxic properties of CAR-T lymphocytes in vivo compared to wild-type CAR-T cells [71]. *Ren* and colleagues used the CRISPR/Cas9 genome editing system to generate allogeneic CAR-T cells with multiple gene knockouts: *TCRα*, *TCRβ*, *B2M*, and *PD-1*. The edited allogeneic CAR-T cells showed enhanced antitumor activity in the NALM6 NSG mouse model without developing GvHD symptoms or graft rejection syndrome [72].

Beijing Zhongshuo Pharmaceutical Technology Development Co. (Beijing, China) has patented a drug based on allogeneic CAR-T lymphocytes with *TRAC* and *B2M* gene knockout using the CRISPR/Cas9 genome editing system (CN116622712A). The authors demonstrated that gene knockout did not affect the cytotoxicity of CAR-T lymphocytes when co-cultured with model tumor cells. A decrease in the expression of the surface marker of T lymphocyte activation CD25 was demonstrated in the co-culture of allogeneic CAR-T cells and allogeneic PBMC compared to CAR-T lymphocytes without knockout in modeling GvHD and graft rejection syndrome in vitro. Allogeneic CAR-T lymphocytes significantly inhibited tumor cell proliferation in vivo and promoted weight maintenance in model mice after administration of Nalm-6 tumor cells. The absence of TCR and MHC-I on the surface of allogeneic CAR-T cells resulted in a significant reduction in GvHD activity and graft rejection syndrome when administered to mice compared to unedited CAR-T lymphocytes [73].

The study by *Chen* and colleagues presents the results of a study on the generation of allogeneic CAR-T lymphocytes with *TRAC* and *B2M* gene knockouts using the CRISPR/Cas9 genome editing system. The authors demonstrated tumor-specific cytotoxicity when co-culturing allogeneic CAR-T lymphocytes with gene knockout and the Raji cell line: secretion of the pro-inflammatory cytokines IFN-γ and TNF-α was comparable in CAR-T lymphocytes with and without gene editing, while in some cases allogeneic CAR-T cells produced more pro-inflammatory cytokines. Allogeneic CAR-T lymphocytes with a gene knockout induced disease remission in mice with tumor cells and prolonged their lifespan. A therapeutic drug based on experimental allogeneic CAR-T cells was developed and its safety was evaluated in patients with B-cell acute lymphoblastic leukemia. Several cases of grade 3 neutropenia and thrombocytopenia, cytokine release syndrome, and infections were reported. However, allogeneic CAR-T cells with *TRAC* and *B2M* genes knockouts did not cause symptoms of neurotoxicity and GvHD [64].

CRISPR Therapeutics (Zug, Switzerland) has patented the allogeneic CAR-T cell drug CARBON (CTX110, NCT04035434) based on the results of the first phase of clinical trials. Allogeneic CAR-T cells knocked out of the *TRAC* and *B2M* genes produced a significant anti-tumor response: 67% of patients (18 out of 27) and 41% of patients (11 out of 27) achieved an overall response rate (the best response from the moment of disease progression) and a complete response (no symptoms of disease), respectively. A complete response was maintained for six months in 19% of patients (5 out of 27), two of whom maintained a complete response for 24 months. However, the most common adverse event associated with the use of allogeneic CAR-T lymphocytes was grade 2 or lower cytokine release syndrome. Neurotoxicity and infections were reported. The absence of GvHD symptoms and progression of side effects with repeated infusions of allogeneic CAR-T cells allows the use of additional doses of these CAR-T lymphocytes to achieve the desired therapeutic effect [74].

## 6. Clinical Trials of Allogeneic NKG2D CAR-T Lymphocytes

The development of allogeneic CAR-T cell manufacturing methods allows the production of universal cellular medicines for the treatment of not only hematological but also solid malignancies, including those expressing NKG2D ligands. Treating solid tumors presents several challenges, including antigen heterogeneity and the limited number of target antigens. Other factors that hinder T cell migration to the tumor site include chemokine dysregulation, abnormal vascularization, and dense tumor stroma [75,76]. NKG2D is a native receptor on the surface of natural killer cells that can recognize multiple ligands on tumor cells. Therefore, NKG2D is considered as a chimeric antigen receptor for tumors expressing NKG2D ligands. NKG2D ligands are present in tumor cells of various origins, including colorectal cancer, ovarian carcinoma, pancreatic cancer, prostate cancer, acute lymphoblastic leukemia, lymphomas and in a number of cancer cell lines, indicating the applicability of NKG2D CAR-T cells for the therapy of hematological and solid tumors [77]. CAR-T therapy targeting NKG2D ligands has been evaluated in patients with advanced solid tumors NCT05302037 [78], NCT06709469 [79].

In the research of *Alexandre Michaux* allogeneic CAR-T cells expressing the natural killer group receptor 2D (NKG2D) were obtained. Along with the expression of NKG2D, allogeneic CAR-T lymphocytes expressed a peptide representing a truncated form of CD3ζ, which allowed blocking the signal from the TCR by inhibiting the activity of the native domain of CD3ζ (TIM). As a result of co-culturing of allogeneic TIM NKG2D CAR-T lymphocytes with PBMC from other donors, a significant decrease in IFN-γ expression was confirmed compared to control alloreactive T lymphocytes. In addition, the absence of the development of the graft-versus-host reaction was confirmed by introducing TIM NKG2D CAR-T lymphocytes into NSG (immunodeficient) mouse models. The authors also obtained two batches of NKG2D CAR-T cells, totaling 48 billion CAR-T cells. Both batches were highly stable and produced a predominantly CD4+ T-cell population with an effector/central memory phenotype and no signs of exhaustion markers. Antitumor cytotoxicity was confirmed by coculturing of allogeneic NKG2D CAR-T lymphocytes with K562 tumor cells: the cells produced more IFN-γ compared to the control group. The cytotoxicity of the experimental cells was also confirmed in vivo. In addition, NKG2D CAR-T cells did not respond to T-cell receptor simulation with the OKT3 activator, confirming the absence of alloreactivity. A bank of clinical-grade allogeneic NKG2D CAR-T cells expressing TIM is being used in the alloSHRINK clinical trial (NCT03692429) [80].

In June 2025, the first phase of clinical trial NCT06709469 started to evaluate the efficacy of allogeneic CAR-T lymphocytes specific for the NKG2D ligand in children, adolescents and young adults with relapsed/refractory T-cell acute lymphoblastic leukemia (r/r T-ALL). The trial will administer allogeneic NKG2D CAR-T lymphocytes at a total dose of up to 3 × 10^6^ cells/kg divided into three infusions. The development of adverse reactions, including CRS and immune effector cell-associated neurotoxicity (ICANS), will be monitored throughout the trial and for five years thereafter. However, there is currently no information on whether additional genomic modifications of allogeneic NKG2D CAR-T cells are planned prior to the NCT06709469 patient trial [79].

A different approach to the production of allogeneic NKG2D CAR-T lymphocytes was used in the ANGELICA clinical trial (NCT05302037) in patients with advanced solid tumors. Instead of using αβ T lymphocytes, which are responsible for the development of GvHD during transplantation from a healthy donor to a patient, the trial used γδ T lymphocytes—a subtype of T cells characterized by the expression of Vγ(2–5, 8 and 9) and Vδ(1–8) chains to form the γδ dimeric receptor. This cell population is less spread than αβ T lymphocytes but is able to recognize antigens in an HLA-independent manner, reducing the risk of developing GvHD. NKG2D γδ CAR-T lymphocytes (CTM-N2D) are derived from healthy donor peripheral blood cells of healthy donors, followed by γδ T cell expansion and mRNA electroporation. The use of CTM-N2D in patients has been carried out with dose escalation: 1 × 10^8^, 3 × 10^8^ and 1 × 10^9^ per infusion, taking into account the patients’ weight. Prior to infusion, patients underwent lymphodepletion with fludarabine and cyclophosphamide. To increase the sensitivity of the tumor cells to treatment and improve the efficacy of CTM-N2D, patients received zoledronic acid and an injection of IL-2. According to the results of the study, the main problem with therapy using NKG2D γδ CAR-T lymphocytes is the recognition of the target ligand on healthy cells. Cases of CRS, ICANS and tumor lysis syndrome have also been reported. However, there is currently no data on the clinical efficacy of allogeneic NKG2D γδ CAR-T lymphocytes, as the final results of the clinical trial NCT05302037 are expected in December 2026 [78].

Thus, as of February 2025, there are a small number of trials for allogeneic NKG2DL-specific CAR-T drugs registered in the ClinicalTrials.gov database (three trials, one of which is paused—NCT04107142 [81] for the treatment of solid tumors compared with allogeneic CAR-T cell drugs for the treatment of hematological tumor diseases. The small number and early stage of clinical trials of NKG2D CAR-T drugs indicate a logical evolution of allogeneic CAR-T cell technology in the transition from hematological to solid tumor as a therapeutic target. However, it is worth mentioning the perspective of using allogeneic CAR-T lymphocytes for the treatment of hematological tumors [82].

## 7. Conclusions

The development of ready-to-use, “off-the-shelf” allogeneic CAR-T cell products has the potential to combine effective cytotoxic activity against tumor cells with improved safety, persistence, and accessibility compared to current autologous approaches. Using healthy donor T cells minimizes the risk of tumor cell contamination during manufacturing, an issue that remains a concern with autologous CAR-T production [83]. Furthermore, unlike patient-derived products, allogeneic CAR-T cells can be generated through standardized, large-scale processes. This enables the creation of cellular banks of universal CAR-T batches, thereby accelerating treatment delivery and reducing delays that are particularly detrimental in rapidly progressing malignancies [82].

The application of this strategy to NKG2D-targeting CAR-T cells represents a promising, yet still emerging, platform for treating solid tumors. Current trials, including alloSHRINK, NCT06709469, and ANGELICA, are investigating distinct strategies such as the use of allogeneic αβ T cells engineered with TCR disruption to prevent GvHD or γδ T cells, which are inherently less prone to alloreactivity. While preclinical studies have confirmed robust cytotoxicity and reduced risk of graft-versus-host responses, clinical data remain preliminary. Early reports underscore both the therapeutic potential and the major challenges that remain, including on-target/off-tumor toxicity due to NKG2D ligand expression on normal tissues, as well as adverse immune-mediated events such as cytokine release syndrome (CRS) and neurotoxicity (ICANS, replacing the outdated “NTS” abbreviation).

The logical trajectory of allogeneic CAR-T cell therapy is its expansion from hematological malignancies, where feasibility has already been demonstrated, into the more complex setting of solid tumors. However, the small number and early developmental stage of NKG2D-focused clinical studies highlight that the field is still in its infancy. Overcoming key hurdles, such as ligand shedding, immunogenic rejection, and limited persistence in solid tumor microenvironments, will be essential before broad clinical translation is achieved.

In summary, continued advances in genome editing, manufacturing optimization, and safety engineering will be critical for translating allogeneic NKG2D CAR-T cells into scalable therapies. Such innovations are expected to significantly expand patient access to next-generation cellular immunotherapies for both hematological and solid tumors.

## Figures and Tables

**Table 2:** Comparative characteristics of site-specific genome editing platforms (ZFN, TALEN, CRISPR/Cas9) applied in allogeneic CAR-T cell engineering [63].

	ZFN	TALEN	CRISPR/Cas9
**Recognition site**	Zinc finger protein	TALE protein	GuideRNA and tracrRNA
**Modification pattern**	Fok1 nuclease	Fok1 nuclease	Cas9 nuclease
**Specificity**	Small number of positional mismatches	Small number of positional mismatches	Positional/multiple consecutive mismatches
**Target sequence size**	9–18 bp	14–20 bp	20 bp- guide + PAM sequence
**Targeting limitations**	Difficult to target non-G-rich sites	5′ targeted base must be a T for each TALEN monomer	Recognizes 3′ G-rich Must precede a PAM sequence of 3–5 nt
**Engineering**	Requires substantial protein engineering	Requires complex molecular cloning methods	Uses standard cloning procedures
**Delivery**	Easy due to small size	Difficult due to large size	Moderate to difficult due to large size of SpCas9
